# Preprocedural Biomarkers of Inflammation and Fibrosis and Echocardiographic Markers of Myocardial Remodeling in New-Onset Conduction Disorders After Transcatheter Aortic Valve Implantation

**DOI:** 10.3390/medicina62091623

**Published:** 2026-08-23

**Authors:** Gordana Bačić, Davorka Lulić, Fabio Kadum, Snježana Hrabrić Vlah, Ivana Smoljan, Vjekoslav Tomulić, Sunčica Buljević, Alen Ružić

**Affiliations:** 1 Clinic for Cardiovascular Diseases, Clinical Hospital Centre Rijeka, Tome Strižića 3, 51000 Rijeka, Croatia; davorkalulic@yahoo.com (D.L.); kadum.fabio@gmail.com (F.K.); ismoljan@yahoo.com (I.S.); vjekoslav.tomulic@uniri.hr (V.T.); alen.ruzic@uniri.hr (A.R.); 2 Department of Internal Medicine, Faculty of Medicine, University of Rijeka, Braće Branchetta 20, 51000 Rijeka, Croatia; 3 Clinical Department of Laboratory Diagnostics, Clinical Hospital Centre Rijeka, Tome Strižića 3, 51000 Rijeka, Croatia; snjezana.hrabric.vlah@kbc-rijeka.hr; 4 Department of Medical Chemistry, Biochemistry and Clinical Chemistry, Faculty of Medicine, University of Rijeka, Braće Branchetta 20, 51000 Rijeka, Croatia; suncica.buljevic@medri.uniri.hr

**Keywords:** transcatheter aortic valve implantation, conduction disorders, interleukin-6, inflammation, C-reactive protein, C-reactive protein-to-albumin ratio, aortic stenosis

## Abstract

*Background and Objectives:* Conduction disorders (CDs) and permanent pacemaker implantation (PPI) remain among the most common complications after transcatheter aortic valve implantation (TAVI). Given their potential impact on long-term outcomes, improved preprocedural identification of patients at high risk for new-onset CDs is increasingly important. We investigated whether selected preprocedural inflammatory and fibrotic biomarkers, along with echocardiographic indices of regional myocardial remodeling, were associated with new-onset CDs after TAVI. *Materials and Methods:* This single-center prospective observational study included 112 patients with severe aortic stenosis undergoing TAVI. Peripheral blood samples were obtained within 24 h before TAVI for measurement of inflammatory and fibrotic biomarkers, including interleukin-6 (IL-6), C-reactive protein (CRP), CRP-to-albumin ratio (CAR), procalcitonin, ferritin, lactate dehydrogenase, and transforming growth factor-β1. The primary outcome was the occurrence of new-onset CDs during the index hospitalization or within three months after TAVI. *Results:* New-onset CDs occurred in 58 patients (51.8%). IL-6 showed the strongest association with the outcome in univariable analysis (OR per 1-SD increase, 9.55; 95% CI 3.00–30.40; *p* < 0.001), remained associated after adjustment for selected clinical and procedural predictors in exploratory models (adjusted OR 10.70; 95% CI 2.43–47.07; *p* = 0.002), and demonstrated the highest, although moderate, discriminatory performance among the evaluated biomarkers (AUC 0.730; 95% CI 0.637–0.823). CRP and CAR were higher in patients with CDs and were significant in univariable analysis, but showed weaker and less consistent adjusted associations. Among echocardiographic markers, AB strain ratio ≥ 2 was the most consistent imaging correlate in exploratory biomarker–echocardiographic models. *Conclusions:* Elevated preprocedural IL-6 was the biomarker most consistently associated with new-onset CDs after TAVI. These findings suggest that higher preprocedural IL-6 levels may reflect patient-specific susceptibility to new-onset CDs after TAVI and could have potential value as an adjunctive biomarker, alongside echocardiographic assessment, for preprocedural risk evaluation, with further validation required in larger prospective studies.

## 1. Introduction

Transcatheter aortic valve implantation (TAVI) is an established therapeutic option for severe aortic stenosis (AS) in patients across all levels of surgical risk [1,2,3,4]. Despite significant improvements in transcatheter heart valve (THV) design and procedural techniques, conduction disorders (CDs) and permanent pacemaker implantation (PPI) remain among the most common complications after TAVI [5]. Reported incidences vary across studies and registries, but new-onset left bundle branch block (LBBB) and PPI occur in approximately 20% and 15% of patients within 30 days after the procedure, respectively, and are associated with increased long-term mortality and heart failure hospitalizations [6,7,8,9,10,11,12]. As TAVI indications expand toward younger and lower-risk populations, improved identification of patients at increased risk for new-onset CDs has become increasingly important [13,14,15].

Although the anatomical proximity between the conduction system (CS) and the aortic valve (AV) may partially explain the occurrence of TAVI-related CDs, emerging evidence suggests that their development results from a complex interplay of anatomical, procedural, and patient-specific factors [16,17,18]. Chronic inflammation and fibrosis play central roles in the pathogenesis of degenerative AS and contribute to structural and electrical myocardial remodeling, potentially involving the CS because of its proximity to the basal septum and periannular region [19,20,21,22,23,24]. Several inflammatory and fibrotic biomarkers, including C-reactive protein (CRP), interleukin-6 (IL-6), tumor necrosis factor-α, and transforming growth factor-β1 (TGF-β1), have been linked to profibrotic molecular mechanisms and imaging-based measures of myocardial fibrosis in patients with AS. However, direct histological evidence in the human CS remains limited [25,26,27,28,29]. Accordingly, patients with severe AS may have clinical or subclinical conduction abnormalities that could increase susceptibility to worsening pre-existing CDs or developing new CDs during or after TAVI [30]. Recent studies have suggested that preprocedural systemic inflammatory biomarkers, including composite indices derived from differential blood counts, may be associated with the risk of TAVI-related CDs and PPI, thereby supporting their potential utility in preprocedural risk assessment [31,32].

Routinely available inflammatory biomarkers, including CRP and the CRP-to-albumin ratio (CAR), have been evaluated mainly as predictors of mortality, rehospitalization, and treatment futility in patients undergoing TAVI [33,34,35]. A broader multimarker approach incorporating procalcitonin (PCT), ferritin, lactate dehydrogenase (LDH), IL-6, and other clinical parameters has also been explored for preprocedural risk stratification in this setting, showing that a greater number of elevated biomarkers was associated with a higher risk of adverse clinical outcomes [36]. However, the association between this broader panel of routinely available and more specific inflammatory and fibrotic biomarkers—including CRP, CAR, PCT, ferritin, LDH, IL-6, and TGF-β1—and new-onset CDs after TAVI has not yet been systematically investigated. Routinely available biomarkers may reflect systemic inflammation and broader patient-specific vulnerability, whereas more specific inflammatory and fibrotic biomarkers, such as IL-6 and TGF-β1, may provide additional mechanistic insight into inflammatory–fibrotic pathways potentially involved in myocardial remodeling and CS vulnerability. In parallel, echocardiographic markers of regional myocardial remodeling may help characterize the structural substrate underlying conduction vulnerability, as previous data suggest that an increased apical-to-basal (AB) strain ratio may be associated with a higher risk of TAVI-related new-onset CDs [37]. Whether these circulating and imaging markers capture related aspects of CS vulnerability in the TAVI setting remains insufficiently clarified.

Therefore, this study aimed to investigate whether selected preprocedural inflammatory and fibrotic biomarkers, along with echocardiographic indices of regional myocardial remodeling, were associated with new-onset CDs in patients undergoing TAVI.

## 2. Materials and Methods

### 2.1. Study Participants

To address this aim, we prospectively screened 198 consecutive patients with severe AS who were admitted for TAVI at the Clinic for Cardiovascular Diseases, Clinical Hospital Centre Rijeka, Croatia, between December 2024 and February 2026. Patients were eligible if they had a confirmed diagnosis of severe AS and were referred for TAVI following comprehensive evaluation by the local Heart Team.

**Figure 1:**
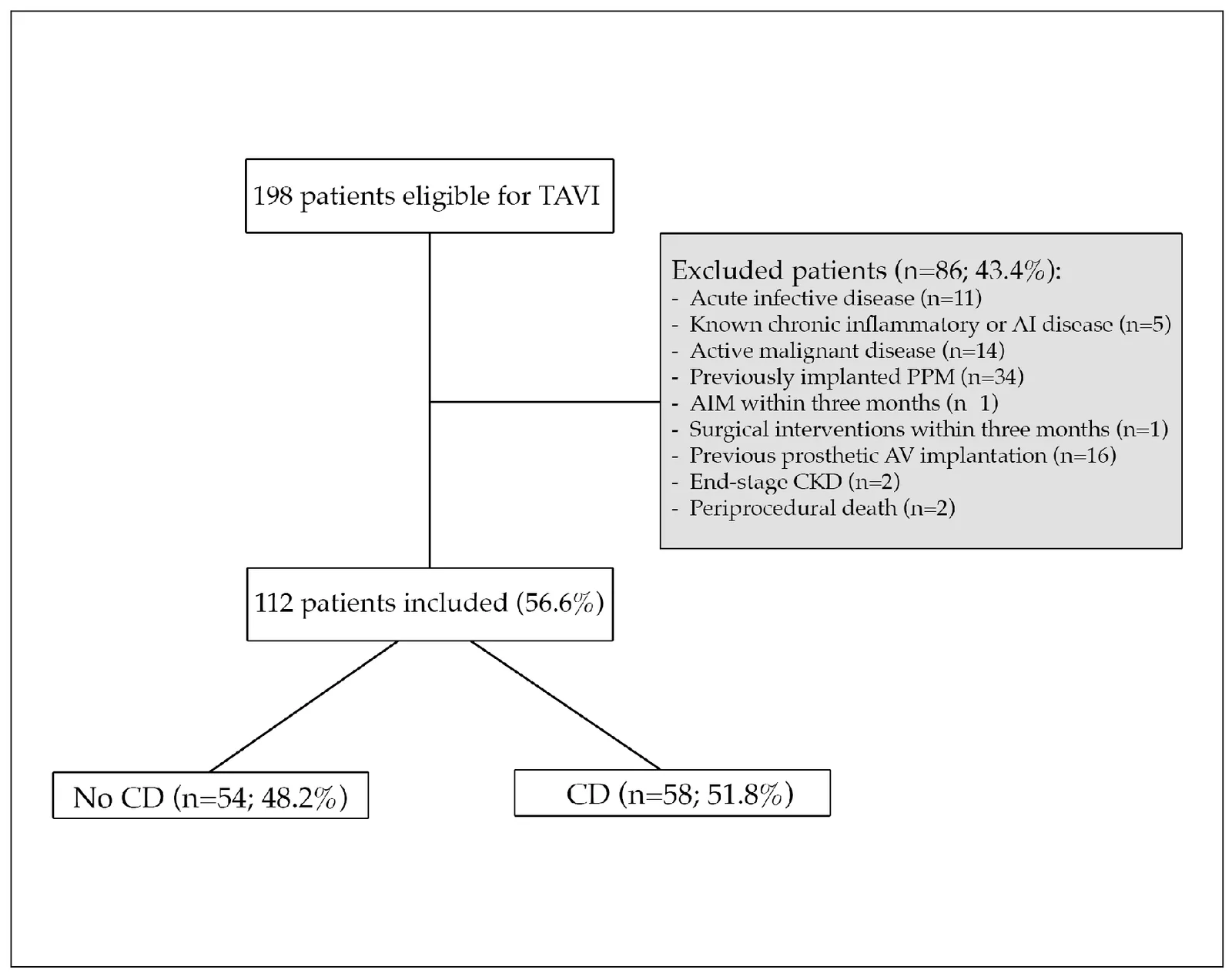
Flowchart of patient selection and reasons for exclusion. Of 198 consecutive patients assessed for eligibility, 112 were included in the final analytic cohort. Two patients died during the procedure before outcome assessment could be completed. All other exclusions were based on baseline eligibility and preprocedural exclusion criteria. Abbreviations: AS, aortic stenosis; TAVI, transcatheter aortic valve implantation; CD, conduction disorder; AI, autoimmune disease; PPM, permanent pacemaker; AMI, acute myocardial infarction; CKD, chronic kidney disease.

Patients were excluded before enrollment if they had a concomitant condition that could affect systemic inflammatory biomarker levels, including chronic inflammatory or autoimmune disease, active or recent malignancy, end-stage chronic kidney disease (eGFR < 15 mL/min), liver dysfunction, corticosteroid or other immunosuppressive therapy, or acute myocardial infarction or surgery within the preceding three months. Patients with acute infection were deferred until clinical resolution. Additional preprocedural exclusion criteria were previous permanent pacemaker implantation and prior surgical or transcatheter aortic valve replacement. After application of baseline eligibility and preprocedural exclusion criteria, patients were prospectively followed during the index hospitalization and for three months after TAVI, including an outpatient visit at the end of this period. Two patients died during the procedure before outcome assessment could be completed and were therefore not included in the final analytic cohort. All remaining exclusions were based on baseline eligibility and preprocedural exclusion criteria, as detailed in Figure 1. The final study cohort included 112 patients.

The required sample size was estimated using G*Power software, version 3.1.9.7, for a two-sided *t*-test comparing two independent groups. Assuming a statistical power of 0.80, an α level of 0.05, and a medium effect size, the calculation indicated that 51 patients were required per group, corresponding to a total minimum sample size of 102 patients. Therefore, the final sample of 112 patients met the estimated requirement for the planned primary between-group comparison, whereas the study was not powered for definitive multivariable modeling, ROC-based threshold estimation, or assessment of incremental predictive value.

The study was conducted in accordance with the ethical principles of the Declaration of Helsinki and was approved by the Institutional Ethics Committee of the Clinical Hospital Centre Rijeka, Rijeka, Croatia, on 25 October 2024 (Class: 003-05/24-01/131; Approval No.: 2170-29-02/1-24-2). Written informed consent was obtained from all participants.

### 2.2. Preprocedural Evaluation

At hospital admission, all patients underwent an initial work-up that included a detailed medical history, physical examination, blood sampling, and 12-lead electrocardiography (ECG) to document baseline heart rhythm and pre-existing CDs. Transthoracic echocardiography (TTE) was performed in all patients, irrespective of prior imaging studies.

#### 2.2.1. Blood Sampling and Biomarker Analysis

Peripheral blood samples were collected at a single time point within 24 h before TAVI as part of the routine preprocedural evaluation, with an additional sample obtained for specific biomarker analyses. Blood was drawn from an antecubital vein into standard VACUETTE^®^ tubes with Sep/Clot Activator or K3EDTA (Greiner Bio-One GmbH, Kremsmünster, Austria), as appropriate. Serum was obtained after centrifugation at 3500 rpm for 10 min. CRP, albumin, PCT, IL-6, ferritin, and LDH were measured immediately in fresh serum, whereas serum aliquots for TGF-β1 analysis were stored at −80 °C until further processing.

Serum concentrations of CRP and ferritin were measured using a turbidimetric method on an AU5800 analyser (Beckman Coulter, Inc., Brea, CA, USA). LDH and albumin levels were measured using a photometric method on the same analyser. PCT was measured using a chemiluminescence immunoassay on a Beckman Coulter DxI 9000 analyser (Beckman Coulter, Inc., Brea, CA, USA). IL-6 was measured using an electrochemiluminescence immunoassay on a Cobas Pro e801 analyser (Roche Diagnostics GmbH, Mannheim, Germany). CAR was calculated as serum CRP level (mg/L) divided by serum albumin level (g/L). Serum TGF-β1 levels were analysed using an enzyme-linked immunosorbent assay with a Human TGF-β1 ELISA Kit (Proteintech Group, Inc., Rosemont, IL, USA; Proteintech Germany GmbH, Planegg-Martinsried, Germany). Optical density was measured using a HiPo MPP-96 microplate photometer (BioSan SIA, Riga, Latvia). All assays were performed according to the manufacturers’ instructions and routine internal laboratory quality-control procedures.

#### 2.2.2. Transthoracic Echocardiography

A comprehensive TTE examination was performed in all patients before TAVI using a Vivid E95 ultrasound system (GE HealthCare, Chicago, IL, USA), following a standard 2D and Doppler protocol in accordance with current echocardiographic recommendations [38]. Echocardiographic data were digitally stored and subsequently analysed offline using commercially available software (EchoPAC version 203; GE Medical Systems, Horten, Norway). Offline strain analyses were performed by an experienced investigator who was blinded to clinical outcomes and biomarker results. Formal intra- and interobserver reproducibility analyses of strain measurements were not performed in the present study. Global longitudinal strain (GLS) was assessed using the speckle-tracking method and reported as absolute values, with higher values indicating better longitudinal systolic function. Segmental longitudinal strain values were further used to derive echocardiographic markers of regional left ventricular (LV) remodeling potentially related to new-onset CDs. Regional longitudinal strain was averaged at the basal, mid-ventricular, and apical LV levels. The apical-to-basal (AB) strain ratio was calculated as mean apical divided by mean basal longitudinal strain; AB strain ratio ≥ 2 was used as a predefined marker of a pronounced difference between apical and basal longitudinal strain values. Relative apical sparing pattern (RASP) was defined as mean apical longitudinal strain divided by the sum of mean basal and midventricular longitudinal strain; RASP > 1 was used as a predefined marker of relative apical sparing.

#### 2.2.3. Multislice Computed Tomography (MSCT)

Preprocedural ECG-gated multislice computed tomography (MSCT), including imaging of the entire aorta and femoral arteries, was performed before the index hospitalization for TAVI. Images were reconstructed and analysed using dedicated software (3mensio Structural Heart version 10.6, Pie Medical Imaging, Maastricht, The Netherlands). Measurements required for procedural planning were obtained in accordance with Society of Cardiovascular Computed Tomography recommendations [39].

### 2.3. Procedure Details

All TAVI procedures were performed according to a standard protocol, under conscious sedation with dexmedetomidine and midazolam, using the transfemoral approach. Valve type and size were selected by implanting operators based on preprocedural MSCT-derived anatomy, annular dimensions, vascular access characteristics, and device availability. The transcatheter heart valve models used in this study included the following balloon-expandable valves: SAPIEN 3 and SAPIEN 3 Ultra RESILIA (Edwards Lifesciences, Irvine, CA, USA) and Myval Octacor (Meril Life Sciences Pvt. Ltd., Vapi, India). Self-expanding valves included Evolut FX and Evolut PRO+ (Medtronic, Minneapolis, MN, USA), and ACURATE neo2 and ACURATE Prime (Boston Scientific, Marlborough, MA, USA). Biomarker results, including IL-6, were not available to the implanting operators at the time of valve selection or procedure planning. Beta-blocker therapy was withheld in all patients 24 h before the procedure. Temporary right ventricular (RV) pacing was used selectively in patients considered at increased risk of new-onset CDs and in those who developed clinically relevant CDs during or after TAVI; it was maintained for at least 24–48 h or until PPI when indicated.

### 2.4. Electrocardiographic Assessment During Hospitalization and Follow-Up

In all patients, 12-lead ECGs were recorded at hospital admission, immediately after TAVI, daily until discharge, and at the three-month follow-up visit. Continuous ECG monitoring was performed during TAVI and for at least 24 h after the procedure in the Intensive Cardiac Care Unit, with extended monitoring in patients with new-onset CDs or an increased risk of delayed high-degree or complete atrioventricular block (HAVB or CAVB). PPI was performed during the index hospitalization when clinically indicated, typically after 24–48 h of persistent CDs or later in cases of delayed severe CDs, in accordance with current recommendations [40]. At the three-month follow-up visit, 12-lead ECG and 24-h Holter monitoring were used to assess late-onset CDs and the evolution of previously documented CDs. Medical records and the hospital information system were also reviewed to identify new CDs, PPI procedures, or other adverse events during the follow-up period.

### 2.5. Study Outcomes

The primary study outcome was defined as the occurrence of clinically relevant new-onset CDs during the index hospitalization or within the three-month follow-up period. These included new-onset LBBB, new-onset right bundle branch block (RBBB), HAVB, and CAVB, defined and further classified according to VARC-3 criteria [41]. PPI, including timing and indication, was recorded descriptively as a relevant consequence of severe, persistent, or high-risk CDs, but was not included as a component of the primary outcome. Although PPI is commonly used as a clinically relevant endpoint in TAVI studies, new-onset CDs were selected as the primary outcome in the present study to capture the broader spectrum of CS vulnerability, including transient and persistent abnormalities that may not necessarily lead to PPI. The limited number of more severe conduction-related events, including HAVB/CAVB and PPI, precluded sufficiently powered subtype-specific or PPI-specific analyses. Therefore, PPI was evaluated only in an exploratory sensitivity analysis. Patients were subsequently categorized into CD and non-CD groups based on the occurrence of the primary outcome, whereas individual CD types, persistence status, and PPI were summarized descriptively.

### 2.6. Statistical Analysis

Statistical analyses were performed to compare patients with and without new-onset CDs after TAVI and to evaluate associations between preprocedural biomarkers, selected echocardiographic parameters, and the primary outcome. Continuous variables were assessed for normality and presented as mean ± standard deviation (SD) or median with interquartile range, as appropriate. Between-group comparisons were performed using Welch’s *t*-test or the Mann–Whitney U test for continuous variables and the chi-square test or Fisher’s exact test for categorical variables, as appropriate.

Logistic regression analyses were performed to evaluate associations between selected variables and new-onset CDs after TAVI. Univariable models were first constructed, and ORs with 95% CIs were reported. Continuous variables were standardized and expressed per one SD increase, except for implantation depth at the non-coronary cusp (ID_NCC_) and membranous septum length (MSL), which were analysed per 1 mm increase. Because CAR had a right-skewed distribution, CAR values were natural log-transformed before standardization; the standardized ln(CAR) variable was used in univariable and multivariable logistic regression analyses. Linearity of IL-6 as a continuous predictor in the logit was assessed using the Box–Tidwell approach by including an IL-6 transformation term and testing its contribution with a likelihood-ratio test.

#### 2.6.1. Exploratory Multivariable Models

Multivariable logistic regression models were constructed as exploratory analyses in two sets. First, separate biomarker-based clinical/procedural models were fitted for IL-6, CRP, and ln(CAR), with each biomarker entered together with four prespecified predictors of TAVI-related CDs: SEV implantation, oversizing > 15%, ID_NCC_, and any pre-existing CD. These covariates were selected a priori based on clinical relevance, prior evidence, and established associations with TAVI-related CDs, while also considering the limited number of outcome events. With 58 outcome events and five predictors per model, the events-per-variable ratio was 11.6; therefore, these models were considered acceptable for exploratory analysis. Second, additional exploratory models combined each biomarker separately with the same four predefined echocardiographic parameters: left ventricular stroke volume index (LV SVi), apical-to-basal (AB) strain ratio ≥ 2, ejection fraction (EF)/basal GLS ratio, and relative apical sparing pattern (RASP) > 1, to explore associations between biomarkers, selected imaging-derived measures of myocardial remodeling, and new-onset CDs. For the IL-6-based clinical/procedural model, three further exploratory assessments were performed: comparison with a biomarker-free reference model, sensitivity analysis using natural log-transformed IL-6, and additional adjustment for natural log-transformed NT-proBNP to address potential residual confounding by baseline disease severity. As an additional exploratory sensitivity analysis focused on PPI, preprocedural IL-6 concentrations were compared between patients with and without PPI, and the association between IL-6 and PPI was evaluated using univariable logistic regression. Owing to the limited number of PPI events, no multivariable PPI model was fitted. All additional exploratory assessments and the PPI-focused sensitivity analysis are presented in the Supplementary Materials.

#### 2.6.2. Model Performance and ROC Analysis

Before multivariable model fitting, multicollinearity was assessed using variance inflation factors (VIFs). All VIFs were low (1.03–1.55) and below the predefined threshold of 5, indicating no relevant multicollinearity. Model performance was summarized using the overall model chi-square test, Nagelkerke R^2^, Hosmer–Lemeshow calibration, classification accuracy, and ROC-derived AUC with 95% CIs. Discriminative performance was compared between models using the DeLong test. Because these models were not developed or validated as clinical prediction tools, performance metrics were interpreted descriptively.

ROC curve analysis was additionally performed for individual biomarkers, with discrimination quantified by AUC and 95% CIs. Data-derived cut-off values were estimated using the Youden index and considered exploratory. No formal correction for multiple testing was applied; therefore, secondary analyses were considered hypothesis-generating. All tests were two-sided, and a *p*-value < 0.05 was considered statistically significant. Statistical analyses were performed using MedCalc^®^ Statistical Software version 22.006 (MedCalc Software Ltd., Ostend, Belgium).

## 3. Results

### 3.1. Study Population and Baseline Characteristics

The final analytic cohort included 112 patients who underwent TAVI for severe symptomatic AS, as shown in Figure 1. During the follow-up period, 58 patients (51.8%) developed new-onset CDs. Patients who developed new-onset CDs were older than those without CDs (median age, 81.0 vs. 78.5 years; *p* = 0.046), and had poorer functional status according to NYHA classification and higher estimated surgical risk. NT-proBNP levels were also significantly higher in patients with new-onset CDs. Sex distribution, comorbidities, baseline rhythm, and mean heart rate were comparable between groups. Pre-existing CDs were numerically more frequent in patients who developed new-onset CDs (41.4% vs. 25.9%; *p* = 0.127), particularly PR interval prolongation, left anterior fascicular block (LAFB), and RBBB, although none of these differences reached statistical significance (Table 1).

### 3.2. Echocardiographic Characteristics and Markers of Myocardial Remodeling

Standard echocardiographic indices of AS severity, LV structure, and LV function were largely comparable between groups (Table 2).

However, patients who developed new-onset CDs had lower Doppler-derived LV SVi, lower basal GLS, and a higher frequency of AB strain ratio ≥ 2 (36 [62.1%] vs. 12 [22.2%] patients; *p* < 0.001). RASP > 1 was also more frequent in the CD group (16 [27.6%] vs. 4 [7.4%]; *p* = 0.006). EF/GLS and EF/basal GLS ratios were higher, while right-sided functional and pulmonary pressure indices also differed, with lower TAPSE and higher PASP in patients with new-onset CDs. In addition, advanced stages of AS-related cardiac damage, particularly stages 3 and 4, were more frequent in the CD group (*p* = 0.004).

### 3.3. Anatomical and Procedural Characteristics

Bicuspid aortic valve morphology and membranous septum length (MSL) were comparable between groups. Compared with the non-CD group, patients with new-onset CDs had a less favorable anatomical and procedural profile, including higher annular and LVOT eccentricity indices, more frequent tapered LVOT morphology, and more frequent self-expanding valve implantation (35 [60.3%] vs. 11 [20.4%]; *p* < 0.001). Implantation depth was greater, particularly at the non-coronary cusp (ID_NCC_ median, 5.0 vs. 3.0 mm; *p* = 0.002). The MSL − ID_NCC_ difference was lower in the CD group, and a negative MSL − ID_NCC_ difference was more frequently observed in this group. Valve oversizing was greater, and oversizing > 15% was also more frequent, in patients with new-onset CDs (24 [41.4%] vs. 5 [9.3%]; *p* < 0.001). The use of pre- or post-dilatation was comparable between groups (Table 3).

### 3.4. Incidence, Timing, and Types of New-Onset Conduction Disorders

Overall, 58 patients (51.8%) developed new-onset CDs during the index hospitalization or within three months after TAVI (Table 4).

As some patients developed more than one CD type, the cumulative number of documented CD types exceeded the number of patients with new-onset CDs. Among the 58 patients with new-onset CDs, 55 (94.8%) had CDs documented during the index hospitalization, whereas three patients (5.2%) had late CDs clinically documented after discharge. Most periprocedural CD events (*n* = 63) occurred immediately after valve implantation (41.8%) or after balloon pre-dilatation (38.2%). Among all documented new-onset CD types (*n* = 66), the most frequent were new-onset LBBB (69.7%) and HAVB or CAVB (25.8%). CDs resolved spontaneously before discharge in 23 patients (39.7%) and persisted until discharge or, in cases of prolonged hospitalization, beyond seven days in 35 patients (60.3%). Among patients with persistent CDs at discharge, 15 (42.9%) still had CDs at the three-month follow-up.

During follow-up, 18 patients (16.1% of the total cohort) underwent PPI, most of them during the index hospitalization and only one after discharge. The main indications were periprocedural CAVB (*n* = 12) and additional ECG changes in patients with pre-existing RBBB or new-onset LBBB, including QRS ≥ 150 ms, PR ≥ 240 ms, or QRS/PR prolongation ≥ 20 ms. The median time from TAVI to PPI was 3 days. Device type was selected by the electrophysiologist according to current recommendations.

### 3.5. Biomarkers and New-Onset Conduction Disorders

Patients in the CD group had higher IL-6 concentrations than those in the non-CD group (median, 5.53 vs. 3.32 ng/L; *p* < 0.001). CRP and CAR were also higher in the CD group (CRP: median, 1.80 vs. 1.20 mg/L; *p* = 0.033; CAR: median, 0.040 vs. 0.026; *p* = 0.034), whereas PCT, ferritin, LDH, and TGF-β1 did not differ significantly between groups (Table 5).

Univariable logistic regression analysis identified IL-6, modeled as a continuous standardized predictor, as the biomarker most strongly associated with new-onset CDs (OR, 9.55; 95% CI, 3.00–30.40; *p* < 0.001). The Box–Tidwell assessment did not provide sufficient evidence to reject the assumption of linearity in the logit for IL-6 (likelihood-ratio χ^2^ = 3.23, df = 1, *p* = 0.072). In comparison, CRP and ln(CAR) were also significantly associated with the outcome, although with smaller effect sizes (CRP: OR, 1.67; 95% CI, 1.06–2.62; *p* = 0.026; ln(CAR): OR, 1.61; 95% CI, 1.08–2.39; *p* = 0.019). PCT, ferritin, LDH, and TGF-β1 were not associated with new-onset CDs. In addition to circulating biomarkers, established procedural and anatomical predictors, including SEV implantation, greater ID_NCC_, valve oversizing > 15%, and tapered LVOT morphology, were also associated with new-onset CDs (Table 6).

In an exploratory sensitivity analysis focused on PPI, preprocedural IL-6 concentrations were numerically higher among patients who underwent pacemaker implantation, but IL-6 was not significantly associated with this endpoint in univariable analysis, with interpretation limited by the small number of events (Table S1).

To assess whether these associations persisted after adjustment, IL-6, CRP, and ln(CAR) were each entered separately with the same four prespecified covariates: SEV implantation, oversizing > 15%, ID_NCC_, and any pre-existing CD (Table 7).

IL-6 remained associated with new-onset CDs after adjustment for these factors (adjusted OR, 10.70; 95% CI, 2.43–47.07; *p* = 0.002), whereas CRP (adjusted OR, 1.53; 95% CI, 0.92–2.60; *p* = 0.120) and ln(CAR) (adjusted OR, 1.52; 95% CI, 0.93–2.51; *p* = 0.098) were no longer associated with the outcome after adjustment. Among the exploratory biomarker-based clinical/procedural models, the IL-6-based model showed numerically higher model-performance metrics, with the highest model χ^2^ and Nagelkerke R^2^, while all three models remained statistically significant overall and showed acceptable calibration (Table S2). Compared with the biomarker-free clinical/procedural reference model, addition of IL-6 suggested an exploratory improvement in model performance, with higher AUC, a significant likelihood-ratio test, and a DeLong comparison that reached nominal statistical significance (Table S3). Findings were also consistent in a sensitivity analysis using natural log-transformed IL-6 in the clinical/procedural model (Table S4). In a sensitivity analysis additionally adjusting the IL-6-based clinical/procedural model for ln(NT-proBNP), IL-6 remained associated with new-onset CDs (Table S5). Among clinical/procedural predictors, SEV implantation remained associated with new-onset CDs across all models after adjustment.

Among individual biomarkers, IL-6 showed the highest, although moderate, discriminatory ability for new-onset CDs (AUC, 0.730; 95% CI, 0.637–0.823; *p* < 0.001), with an exploratory Youden index-derived cut-off of 4.88 ng/L yielding a sensitivity of 60.3% and a specificity of 81.5%. CRP and CAR showed weaker discriminatory performance, whereas PCT, LDH, ferritin, and TGF-β1 had AUC values between 0.50 and 0.58, indicating limited discriminatory ability for new-onset CDs (Table 8).

**Figure 2:**
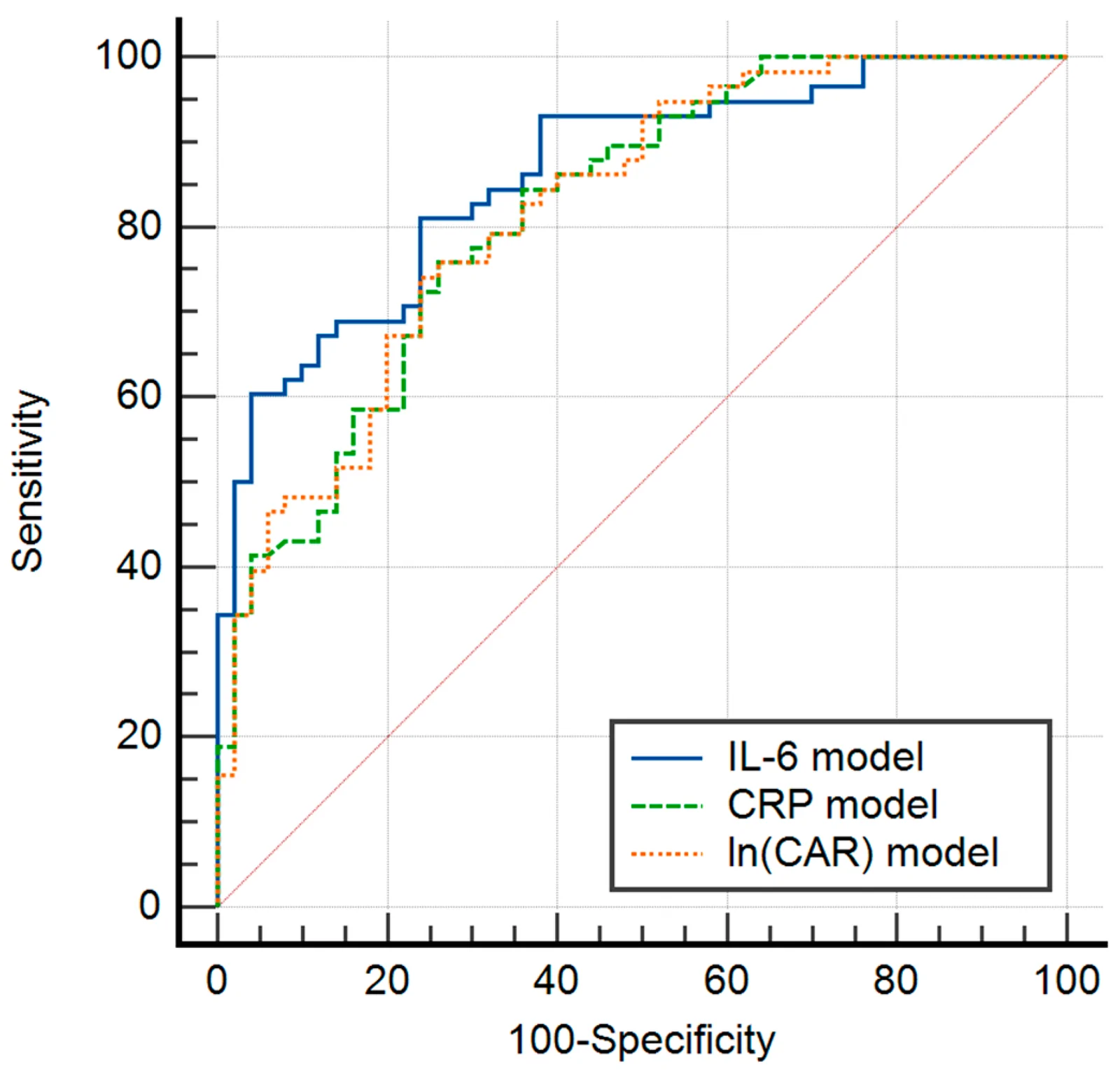
Receiver operating characteristic (ROC) curves of the IL-6-, CRP-, and ln(CAR)-based exploratory clinical/procedural models for new-onset conduction disorders after TAVI.

At the model level, ROC curves for the IL-6-, CRP-, and ln(CAR)-based clinical/procedural models are shown in Figure 2, with detailed model-performance metrics and pairwise DeLong comparisons provided in Tables S2 and S6.

### 3.6. Echocardiographic Markers and New-Onset Conduction Disorders

In univariable logistic regression, AB strain ratio ≥ 2 and RASP > 1 showed the strongest echocardiographic associations with new-onset CDs (AB strain ratio ≥ 2: OR, 5.73; 95% CI, 2.49–13.17; *p* < 0.001; RASP > 1: OR, 4.76; 95% CI, 1.48–15.34; *p* = 0.009). Other significant markers were EF/basal GLS (OR, 1.86; 95% CI, 1.13–3.07; *p* = 0.015), EF/GLS (OR, 1.58; 95% CI, 1.03–2.41; *p* = 0.034), and AS-related cardiac damage stage (OR, 1.72; 95% CI, 1.08–2.73; *p* = 0.023). As GLS was reported as an absolute value, higher basal GLS indicated better basal longitudinal function and was inversely associated with new-onset CDs (OR, 0.57; 95% CI, 0.38–0.86; *p* = 0.007) (Table 9).

In exploratory biomarker–echocardiographic (ECHO) models, IL-6 remained the biomarker most strongly associated with new-onset CDs (adjusted OR, 9.08; 95% CI, 2.47–33.30; *p* = 0.001). CRP was also significant in the CRP + ECHO model (adjusted OR, 1.70; 95% CI, 1.03–2.83; *p* = 0.039), whereas ln(CAR) showed a borderline association (adjusted OR, 1.59; 95% CI, 0.99–2.55; *p* = 0.055). Among echocardiographic variables, only AB strain ratio ≥ 2 remained consistently associated across models (adjusted OR range, 4.12–5.13) (Table 10).

**Figure 3:**
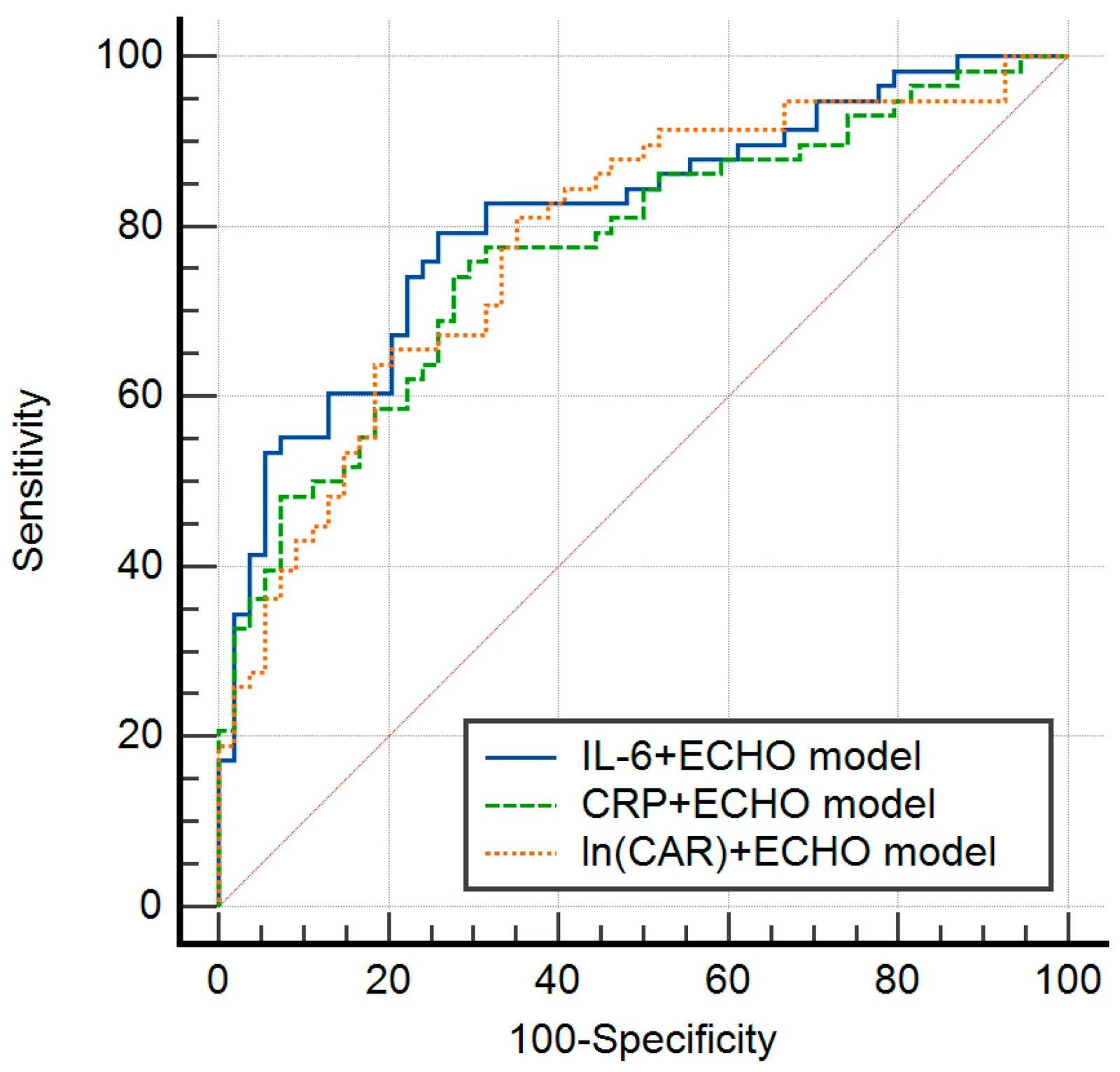
Receiver operating characteristic (ROC) curves of the IL-6 + ECHO, CRP + ECHO, and ln(CAR) + ECHO exploratory models for new-onset conduction disorders after TAVI.

Model performance was numerically the highest for the exploratory IL-6 + ECHO model; however, because the ECHO models were not directly compared with a corresponding biomarker-free clinical/procedural reference model, these findings were interpreted descriptively and not as evidence of incremental predictive value. All models were statistically significant overall (*p* < 0.001), showed acceptable calibration, and had comparable discrimination, with no significant DeLong differences (Tables S7 and S8). The ROC curves of the IL-6 + ECHO, CRP + ECHO, and ln(CAR) + ECHO models are shown in Figure 3.

## 4. Discussion

In this study, elevated preprocedural IL-6 showed the most consistent association with new-onset CDs after TAVI, whereas higher CRP and CAR values showed weaker and less consistent associations. Recent evidence suggests that the development of post-TAVI CDs may involve not only procedure-related mechanical injury but also a combination of mechanical stress and a postprocedural inflammatory response [42,43]. This concept is further supported by observational and early interventional studies in which anti-inflammatory strategies, such as colchicine or perioperative glucocorticoids, have been investigated to attenuate postprocedural inflammation, with preliminary evidence suggesting a possible reduction in new-onset CDs, although this requires confirmation in larger prospective studies [44,45,46].

The present study focused on preprocedural systemic inflammatory and fibrotic biomarkers as potential indicators of pre-existing CS vulnerability and increased susceptibility to new-onset CDs after TAVI. In this context, the degree of preprocedural inflammation may represent a systemic signal of a broader pathological substrate involving myocardial and possibly conduction tissue fibrosis. Shi and colleagues reported that preprocedural systemic inflammatory indices, including the systemic immune-inflammation index (SII) and differential blood count-derived ratios, were associated with post-TAVI conduction block and proposed that inflammation-related CS vulnerability may be mediated by innate immune activation, oxidative stress, and fibrotic remodeling [32]. Another study found that a higher preprocedural neutrophil-to-lymphocyte ratio (NLR) was associated with subsequent PPI after TAVI, particularly among patients receiving SEVs, supporting the potential relevance of systemic inflammation in this setting [33].

To the best of our knowledge, the present study is the first to evaluate a panel of preprocedural systemic inflammatory and fibrotic biomarkers, including both routinely available markers and more specific biomarkers such as IL-6 and TGF-β1, in relation to new-onset CDs after TAVI. Among the evaluated biomarkers, IL-6 showed the strongest association with new-onset CDs after adjustment for selected clinical and procedural predictors, and demonstrated the highest, although moderate, discriminatory performance among the evaluated biomarkers in ROC analysis (AUC = 0.730). This association is biologically consistent with the established role of IL-6 in myocardial remodeling and fibrogenesis in AS, as IL-6 has been shown to promote fibroblast activation and extracellular matrix deposition [27,28,30], as well as inflammation-related electrophysiological remodeling [47,48]. In our cohort, higher IL-6 levels may therefore reflect a more pronounced inflammatory–fibrotic phenotype in patients with AS, commonly involving the basal LV segments and periannular region adjacent to key CS structures [49]. Although direct histological evidence linking IL-6 specifically to fibrosis of the atrioventricular node or His–Purkinje system in humans remains limited, IL-6-related fibrotic and electrophysiological remodeling within the basal septal and periannular substrate could contribute to CS vulnerability and lower the threshold for clinically evident CDs when additional mechanical stress is imposed during TAVI [24,31,47,48].

The echocardiographic findings in our cohort further support this interpretation, as patients who developed new-onset CDs had lower basal GLS and a higher prevalence of an AB strain ratio ≥ 2, consistent with more pronounced regional myocardial remodeling [50,51]. Importantly, IL-6 remained associated with new-onset CDs after adjustment for selected established clinical and procedural predictors. Sensitivity analyses using natural log-transformed IL-6, as well as additional adjustment for natural log-transformed NT-proBNP, yielded similar findings, supporting the consistency of the observed association across exploratory analyses. By contrast, the exploratory PPI-focused sensitivity analysis did not show a significant association between IL-6 and PPI, supporting the interpretation that the primary composite outcome captured a broader and more heterogeneous spectrum of CS vulnerability than PPI alone. Together, these results suggest that IL-6 may capture a component of risk not fully explained by established clinical and procedural predictors or by baseline disease severity, although residual confounding by a more advanced clinical phenotype cannot be excluded. Accordingly, elevated preprocedural IL-6 may reflect a pre-existing inflammatory–fibrotic substrate that could increase CS susceptibility to additional mechanical stress during TAVI, resulting in clinically evident CDs.

From a clinical perspective, preprocedural IL-6 assessment may provide an additional signal of susceptibility to new-onset CDs after TAVI and could complement established clinical, electrocardiographic, anatomical, procedural, and echocardiographic markers. However, the moderate discriminatory performance of IL-6, the limited sample size, the data-driven nature of the Youden-derived threshold, and the absence of internal or external validation preclude its interpretation as a clinically applicable cut-off or its immediate use for procedural planning, postprocedural ECG monitoring, or discharge decisions. Future studies should determine whether IL-6 provides clinically relevant complementary information alongside established predictors and validated risk models, and whether its integration into multiparametric preprocedural assessment can refine individualized risk evaluation for new-onset CDs after TAVI.

CRP and CAR showed a broadly similar but weaker pattern of association. Both markers were higher in patients who developed new-onset CDs and were significantly associated with the outcome in univariable analysis, but their associations were attenuated after adjustment for established clinical and procedural predictors. CRP did not remain associated in the clinical/procedural model after adjustment, but retained a modest association in the exploratory CRP + ECHO model, whereas ln(CAR) showed only a borderline association in the ln(CAR) + ECHO model. Although population-based studies have reported an independent dose–response relationship between elevated high-sensitivity CRP (hsCRP) levels and incident CDs, this apparent discrepancy may be explained by differences in clinical and pathophysiological context [52]. Whereas CRP-based inflammatory markers may reflect chronic low-grade inflammatory exposure contributing to incident CDs in the general population, new-onset CDs after TAVI are more likely driven by the interaction between pre-existing CS vulnerability and procedure-related mechanical stress. Thus, in the present cohort, CRP and CAR may have identified patients with a higher systemic inflammatory burden and greater biological vulnerability, but their adjusted contribution was less consistent than that of IL-6 after adjustment for established TAVI-related determinants. Therefore, CRP and CAR may help characterize systemic inflammatory burden, but appear to provide less adjusted information than IL-6 for preprocedural risk assessment of new-onset CDs after TAVI.

Despite its established role in fibrotic signaling, serum total TGF-β1 was not associated with new-onset CDs in the present cohort [53]. This finding should be interpreted cautiously because circulating total TGF-β1 includes latent forms and may not accurately reflect locally active profibrotic signaling within the basal septum or CS [54,55]. In addition, preanalytical variability, serum-based ELISA quantification, binding-protein interactions, a single preprocedural measurement, and limited sample size may have reduced the ability to detect an association [56]. Thus, the lack of an association between serum total TGF-β1 and new-onset CDs does not exclude a role of TGF-β1-mediated fibrosis in CS vulnerability, but suggests that circulating total TGF-β1 may be an insufficient surrogate marker in this setting.

The remaining biomarkers evaluated, including PCT, LDH, and ferritin, did not differ significantly between groups and were not associated with new-onset CDs in univariable analysis. This may reflect their limited specificity for the chronic inflammatory–fibrotic substrate or CS vulnerability relevant to TAVI-related CDs, particularly in a clinically stable preprocedural population in whom acute infections, chronic inflammatory or autoimmune diseases, active malignancy, and other conditions that markedly affect systemic inflammatory biomarker levels had been excluded [57,58]. Conversely, this strict patient selection supports the interpretation that the observed IL-6, CRP, and CAR values more likely reflected an AS-related inflammatory state than unrelated inflammatory, infectious, or malignant conditions, thereby supporting their potential relevance to the vulnerable substrate associated with new-onset CDs after TAVI.

Beyond basal GLS and AB strain ratio ≥ 2, several echocardiographic markers reflecting basal or global LV remodeling were associated with new-onset CDs in univariable analysis, including RASP > 1, EF/basal GLS, EF/GLS, and AS-related cardiac damage stage. Among these variables, AB strain ratio ≥ 2 was the most consistent echocardiographic marker, remaining associated with the outcome across the exploratory biomarker–echocardiographic models. These results are in line with previous data showing that an AB strain ratio ≥ 2 is associated with a higher risk of TAVI-related new-onset CDs [37]. They also support the concept that regional myocardial remodeling, particularly involving the basal LV segments and periannular region adjacent to the CS, may reflect a vulnerable myocardial substrate susceptible to procedure-related mechanical injury. The associations of basal GLS, RASP, and AS-related cardiac damage with new-onset CDs should be interpreted as supportive and hypothesis-generating, as they were mainly observed in univariable analyses. Moreover, the biomarker–echocardiographic models showed comparable discrimination and did not clearly outperform the IL-6-based clinical/procedural model. Overall, selected echocardiographic markers may complement biomarker assessment, but their incremental value for risk prediction requires validation in larger cohorts.

The fact that most CDs occurred during the procedure itself supports mechanical stress as an immediate trigger, whereas the association with baseline inflammatory biomarkers suggests that this trigger may act on a pre-existing inflammatory–fibrotic substrate. The persistence of CDs until discharge in 60.3% of patients with new-onset CDs may further indicate that this substrate contributes not only to CD occurrence but also to CD persistence, although this requires confirmation in dedicated analyses. Postprocedural inflammation may contribute to delayed CDs, but is less likely to be the dominant mechanism underlying immediate procedure-related events.

Established anatomical and procedural predictors, including SEV implantation, greater implantation depth, valve oversizing, and tapered LVOT morphology, were also associated with new-onset CDs. This supports the internal consistency of our findings and indicates that the association between IL-6 and new-onset CDs occurred within the expected procedural risk framework rather than in isolation.

The relatively high incidence of new-onset CDs in our cohort likely reflects the broad VARC-3-based outcome definition, which captured clinically relevant conduction abnormalities beyond PPI, including both transient and persistent events, together with systematic in-hospital ECG surveillance and follow-up assessment. This outcome definition was intentionally selected to evaluate CS vulnerability rather than PPI alone.

### Study Limitations

Several limitations should be acknowledged. This was a single-center study with a relatively small and strictly selected cohort, which may limit the generalizability of the findings to broader real-world TAVI populations. Exclusion of patients who died before complete outcome assessment may have introduced selection bias, particularly because the primary endpoint could occur during the index hospitalization and most observed CDs were periprocedural. Although the sample size was adequate for the prespecified between-group comparison, it was limited for definitive multivariable modeling, discrimination analyses, and assessment of incremental predictive value. The limited number of outcome events restricted the number of covariates that could be included in each exploratory model, and adjustment for all potentially relevant predictors was therefore not feasible. Because this study was designed to evaluate associations rather than to develop a clinical prediction model, no formal internal or external validation was performed; therefore, ROC-derived AUCs, calibration results, DeLong comparisons, and biomarker cut-offs should be interpreted as descriptive and hypothesis-generating. No formal correction for multiple comparisons was applied, increasing the possibility of type I error.

Although the prospective design and consecutive patient inclusion may have reduced selection bias, the observational nature of the study precludes causal inference. The composite primary outcome included different types and severities of conduction abnormalities, which may have heterogeneous mechanisms and clinical implications. Because the numbers of PPI and HAVB/CAVB were limited, the study was not powered for reliable subtype-specific analyses. In addition, circulating biomarkers and echocardiographic parameters are indirect markers and cannot directly confirm local inflammatory or fibrotic remodeling within the basal septum or CS. No formal intra- or interobserver reproducibility analysis was performed for strain-derived parameters. Finally, postprocedural CDs were assessed using continuous ECG monitoring during the first 24 h after TAVI, serial 12-lead ECGs during hospitalization, and 24-h Holter monitoring at follow-up; therefore, intermittent or asymptomatic late CDs may not have been fully captured.

## 5. Conclusions

In this prospective single-center study, elevated preprocedural IL-6 showed the most consistent association with new-onset CDs after TAVI and remained associated after adjustment for selected clinical, procedural, and imaging predictors, whereas CRP and CAR showed weaker and less consistent associations. Selected echocardiographic markers of regional LV remodeling, particularly AB strain ratio ≥ 2, supported the presence of a vulnerable myocardial substrate but did not clearly improve discrimination beyond the IL-6-based clinical/procedural model. These findings suggest that IL-6 may reflect a patient-specific inflammatory–fibrotic component of susceptibility to TAVI-related CDs that is not fully explained by established clinical, procedural, or imaging-derived markers. However, given the exploratory design, limited sample size, moderate discriminatory performance, and absence of formal validation, larger multicenter studies are needed to confirm these findings and to determine whether IL-6, alone or integrated with imaging markers, provides clinically meaningful incremental information beyond established preprocedural predictors and validated risk models.

## Figures and Tables

**Table 1:** Baseline demographic, clinical, laboratory, and electrocardiographic characteristics according to new-onset conduction disorders after TAVI.

Variable	Overall (N = 112)	No CD (N = 54)	CD (N = 58)	*p*-Value
Demographic, clinical, and laboratory characteristics				
Age, years	80.0 (76.0–83.2)	78.5 (74.0–82.0)	81.0 (77.2–84.0)	**0.046**
Male sex, *n* (%)	56 (50.0%)	30 (55.6%)	26 (44.8%)	0.344
BMI, kg/m^2^	27.6 (24.4–29.9)	26.9 (24.1–29.4)	28.0 (24.6–30.1)	0.108
STS PROM, %	2.78 (1.80–4.78)	2.48 (1.44–4.21)	3.30 (1.98–5.44)	**0.037**
EuroSCORE II, %	2.30 (1.45–3.72)	2.06 (1.37–3.02)	2.85 (1.73–3.88)	**0.038**
NYHA functional class, *n* (%)				**0.005**
NYHA I	8 (7.1%)	6 (11.1%)	2 (3.4%)	
NYHA II	70 (62.5%)	40 (74.1%)	30 (51.7%)	
NYHA III	33 (29.5%)	8 (14.8%)	25 (43.1%)	
NYHA IV	1 (0.9%)	0 (0.0%)	1 (1.7%)	
Hypertension, *n* (%)	106 (94.6%)	51 (94.4%)	55 (94.8%)	1.000
Hyperlipidaemia, *n* (%)	99 (88.4%)	47 (87.0%)	52 (89.7%)	0.891
Diabetes mellitus, *n* (%)	38 (33.9%)	19 (35.2%)	19 (32.8%)	0.943
Coronary artery disease, *n* (%)	41 (36.6%)	24 (44.4%)	17 (29.3%)	0.143
Heart failure, *n* (%)	58 (51.8%)	26 (48.1%)	32 (55.2%)	0.579
Atrial fibrillation/flutter, *n* (%)	27 (24.1%)	11 (20.4%)	16 (27.6%)	0.502
Hemoglobin, g/L	133.06 ± 19.33	134.33 ± 19.26	131.88 ± 19.50	0.504
eGFR, mL/min/1.73 m^2^	65.00 (46.50–81.00)	69.50 (49.50–84.75)	60.00 (45.50–74.00)	0.078
NT-proBNP, ng/L	732.50 (314.50–1771.75)	461.50 (273.50–1299.50)	849.00 (404.50–2974.00)	**0.006**
Electrocardiographic characteristics				
Sinus rhythm, *n* (%)	99 (88.4%)	50 (92.6%)	49 (84.5%)	0.297
Heart rate, bpm	69.0 (60.0–77.0)	67.0 (59.2–76.0)	70.5 (63.0–77.0)	0.108
PR interval, ms	171.0 (160.0–198.0)	170.0 (160.0–196.5)	180.0 (160.0–200.0)	0.836
QRS duration, ms	100.0 (85.0–109.2)	95.5 (84.0–104.0)	100.0 (85.2–109.8)	0.397
Any pre-existing CD, *n* (%) *	38 (33.9%)	14 (25.9%)	24 (41.4%)	0.127
LBBB	3 (2.7%)	2 (3.7%)	1 (1.7%)	0.608
RBBB	9 (8.0%)	3 (5.6%)	6 (10.3%)	0.492
LAFB	15 (13.4%)	5 (9.3%)	10 (17.2%)	0.082
PR ≥ 200 ms	20 (17.9%)	8 (14.8%)	12 (20.7%)	0.414

Continuous variables are presented as mean ± standard deviation or median (interquartile range), as appropriate; categorical variables are presented as *n* (%). Statistically significant *p*-values are shown in bold. * “Any pre-existing CD” refers to patients with at least one pre-existing conduction disorder; individual conduction disorder types are not mutually exclusive. Abbreviations: BMI, body mass index; CD, conduction disorder; eGFR, estimated glomerular filtration rate; LAFB, left anterior fascicular block; LBBB, left bundle branch block; NT-proBNP, N-terminal pro-B-type natriuretic peptide; NYHA, New York Heart Association; RBBB, right bundle branch block; STS PROM, Society of Thoracic Surgeons Predicted Risk of Mortality; TAVI, transcatheter aortic valve implantation.

**Table 2:** Baseline echocardiographic parameters according to new-onset conduction disorders after TAVI.

Variable	Overall (N = 112)	No CD (N = 54)	CD (N = 58)	*p*-Value
Conventional parameters				
AV Vmax, m/s	4.20 (4.00–4.53)	4.20 (4.00–4.60)	4.25 (4.04–4.50)	0.607
AV PG mean, mmHg	45.50 (40.00–53.75)	45.50 (40.51–51.98)	45.30 (38.95–53.98)	0.868
AVAi, cm^2^/m^2^	0.44 ± 0.10	0.46 ± 0.10	0.43 ± 0.10	0.140
LV EF, %	62.00 (56.75–66.00)	62.00 (57.00–65.75)	61.00 (56.25–65.75)	0.576
LV SVi (mL/m^2^)	47.46 ± 11.34	50.04 ± 11.95	45.06 ± 10.27	**0.020**
LV mass index, g/m^2^	122.75 (110.45–141.30)	125.60 (108.80–140.95)	119.45 (110.93–141.20)	0.937
TAPSE, mm	21.48 ± 3.68	22.20 ± 3.92	20.81 ± 3.34	**0.046**
PASP, mmHg	31.05 (23.00–37.67)	29.05 (22.85–36.00)	33.80 (23.50–43.75)	**0.040**
Myocardial deformation parameters				
GLS, %	17.10 ± 4.09	17.73 ± 3.93	16.51 ± 4.17	0.114
Basal GLS, %	12.18 ± 3.14	13.04 ± 3.18	11.38 ± 3.02	**0.005**
AB strain ratio	1.95 (1.54–2.16)	1.72 (1.54–1.98)	2.01 (1.67–2.37)	**0.017**
AB strain ratio ≥ 2	48 (42.9%)	12 (22.2%)	36 (62.1%)	**<0.001**
RASP > 1	20 (17.9%)	4 (7.4%)	16 (27.6%)	**0.006**
EF/GLS ratio	3.50 (3.05–3.91)	3.30 (2.95–3.64)	3.69 (3.23–3.98)	**0.009**
EF/basal GLS ratio	4.95 (4.20–5.88)	4.54 (3.90–5.50)	5.05 (4.70–5.98)	**0.004**
AS-related cardiac damage stage				**0.004**
Stage 0	3 (2.7%)	0 (0.0%)	3 (5.2%)	
Stage 1	7 (6.2%)	5 (9.3%)	2 (3.4%)	
Stage 2	69 (61.6%)	41 (75.9%)	28 (48.3%)	
Stage 3	18 (16.1%)	5 (9.3%)	13 (22.4%)	
Stage 4	15 (13.4%)	3 (5.6%)	12 (20.7%)	

Data are presented as mean ± standard deviation, median (interquartile range), or *n* (%), as appropriate. GLS values are reported as absolute values. Statistically significant *p*-values are shown in bold. Abbreviations: AB, apical-to-basal; AS, aortic stenosis; AV, aortic valve; AVAi, indexed aortic valve area; CD, conduction disorder; EF, ejection fraction; GLS, global longitudinal strain; LV, left ventricular; PASP, pulmonary artery systolic pressure; PG, pressure gradient; RASP, relative apical sparing pattern; SVi, stroke volume index; TAPSE, tricuspid annular plane systolic excursion; Vmax, maximum velocity.

**Table 3:** Anatomical and procedural characteristics according to new-onset conduction disorders after TAVI.

Variable	Overall (N = 112)	No CD (N = 54)	CD (N = 58)	*p*-Value
Bicuspid aortic valve	11 (9.8%)	5 (9.3%)	6 (10.3%)	0.535
MSL, mm	4.47 ± 1.58	4.61 ± 1.69	4.34 ± 1.47	0.363
Annulus eccentricity index	0.22 ± 0.07	0.21 ± 0.08	0.24 ± 0.06	**0.021**
LVOT eccentricity index	0.27 ± 0.08	0.26 ± 0.08	0.29 ± 0.07	**0.017**
Tapered LVOT	10 (8.9%)	1 (1.9%)	9 (15.5%)	**0.037**
ID_NCC_, mm	4.00 (3.00–6.00)	3.00 (2.00–4.75)	5.00 (3.00–6.00)	**0.002**
ID_LCC_, mm	5.00 (3.00–6.00)	3.00 (2.00–5.00)	5.00 (4.00–6.00)	**<0.001**
MSL − ID_NCC_, mm	0.43 ± 2.99	1.31 ± 2.90	−0.39 ± 2.87	**0.002**
MSL − ID_NCC_				**0.019**
MSL − ID_NCC_ < 0	50 (44.6%)	18 (33.3%)	32 (55.2%)	
MSL − ID_NCC_ ≥ 0	62 (55.4%)	36 (66.7%)	26 (44.8%)	
Oversizing, %	9.70 (4.51–15.93)	7.95 (4.06–12.98)	12.15 (5.14–17.55)	**0.011**
Oversizing >15%	29 (25.9%)	5 (9.3%)	24 (41.4%)	**<0.001**
Valve type				**<0.001**
Self-expanding valve	46 (41.1%)	11 (20.4%)	35 (60.3%)	
Balloon-expandable valve	66 (58.9%)	43 (79.6%)	23 (39.7%)	
Valve size, mm	26.00 (24.12–29.00)	26.00 (23.38–27.50)	26.00 (24.50–29.00)	0.116
Pre-dilatation	81 (72.3%)	35 (64.8%)	46 (79.3%)	0.133
Post-dilatation	9 (8.0%)	4 (7.4%)	5 (8.6%)	1.000

Data are presented as mean ± standard deviation, median (interquartile range), or *n* (%), as appropriate. Statistically significant *p*-values are shown in bold. Abbreviations: CD, conduction disorder; ID_LCC_, implantation depth at the left coronary cusp; ID_NCC_, implantation depth at the non-coronary cusp; LVOT, left ventricular outflow tract; MSL, membranous septum length; TAVI, transcatheter aortic valve implantation.

**Table 4:** Study outcomes related to new-onset conduction disorders and permanent pacemaker implantation.

Outcome	*n* (%)
Patients with new-onset CDs	58 (51.8%)
During hospitalization (periprocedural CD)	55 (94.8%)
During follow-up (late CD)	3 (5.2%)
Timing of periprocedural new-onset CD events (*n* = 63)	
Procedural CD (<24 h)	55 (87.3%)
Before wire crossing	2 (3.6%)
After wire crossing	9 (16.4%)
After balloon pre-dilatation	21 (38.2%)
After valve implantation	23 (41.8%)
Delayed CD (>24 h)	8 (12.7%)
Documented new-onset CD types (*n* = 66)	
CAVB/HAVB	17 (25.8%)
LBBB	46 (69.7%)
RBBB	3 (4.5%)
Clinical course among patients with new-onset CD (*n* = 58)	
Transient CD	23 (39.7%)
Persistent CD	35 (60.3%)
Persistent CD at 3-month follow-up	15 (42.9%)
Permanent pacemaker implantation (PPI)	18 (16.1%)
PPI during hospitalization	17 (94.4%)
PPI during follow-up	1 (5.6%)
Indication for PPI (*n* = 18)	
CAVB/HAVB	12 (66.7%)
New-onset LBBB with additional CD	3 (16.7%)
New-onset or pre-existing RBBB with additional CD	3 (16.7%)
Time to PPI, days	3.00 (1.00–4.00)

Data are presented as *n* (%) or median (interquartile range), as appropriate. CDs were defined and classified according to VARC-3 criteria [41]. Percentages were calculated using the denominator indicated in each section: all patients with new-onset CDs (*n* = 58), periprocedural CD events (*n* = 63), documented CD types (*n* = 66), patients with persistent CD at discharge (*n* = 35), or patients who received PPI (*n* = 18), as applicable. Abbreviations: CAVB, complete atrioventricular block; CD, conduction disorder; HAVB, high-grade atrioventricular block; LBBB, left bundle branch block; PPI, permanent pacemaker implantation; RBBB, right bundle branch block; VARC-3, Valve Academic Research Consortium-3.

**Table 5:** Preprocedural biomarker levels according to new-onset conduction disorders after TAVI.

Variable	Overall (N = 112)	No CD (N = 54)	CD (N = 58)	*p*-Value
CRP (mg/L)	1.55 (0.90–2.90)	1.20 (0.80–2.20)	1.80 (0.93–3.27)	**0.033**
CAR	0.034 (0.020–0.069)	0.026 (0.019–0.056)	0.040 (0.022–0.081)	**0.034**
IL-6 (ng/L)	3.98 (3.06–6.07)	3.32 (2.67–4.24)	5.53 (3.24–7.41)	**<0.001**
PCT (µg/L)	0.03 (0.02–0.04)	0.03 (0.02–0.04)	0.03 (0.02–0.04)	0.932
Ferritin (µg/L)	69.50 (33.75–158.50)	77.50 (33.50–153.50)	68.00 (34.00–162.50)	0.643
LDH (U/L)	186.00 (163.00–219.50)	182.50 (157.00–214.25)	191.00 (169.25–224.25)	0.141
TGF-β1 (pg/mL)	14,592 (12,616–16,300)	14,366 (12,197–16,203)	14,679 (12,989–16,430)	0.487

Data are presented as median (interquartile range). Statistically significant *p*-values are shown in bold. Abbreviations: CAR, C-reactive protein-to-albumin ratio; CRP, C-reactive protein; IL-6, interleukin-6; LDH, lactate dehydrogenase; PCT, procalcitonin; TGF-β1, transforming growth factor-β1.

**Table 6:** Univariable logistic regression analysis of variables evaluated for association with new-onset conduction disorders after TAVI.

Variable	OR	95% CI	*p*-Value
Inflammatory and fibrotic biomarkers			
CRP (mg/L), per 1-SD	1.67	1.06–2.62	**0.026**
ln(CAR), per 1-SD	1.61	1.08–2.39	**0.019**
IL-6 (ng/L), per 1-SD	9.55	3.00–30.40	**<0.001**
PCT (µg/L), per 1-SD	0.95	0.65–1.38	0.784
Ferritin (µg/L), per 1-SD	0.78	0.51–1.19	0.246
LDH (U/L), per 1-SD	1.45	0.94–2.22	0.091
TGF-β1 (pg/mL), per 1-SD	0.69	0.47–1.03	0.069
Clinical, anatomical, and procedural parameters			
Age, years	1.06	0.99–1.14	0.082
Male sex	0.65	0.31–1.37	0.257
Diabetes mellitus	0.90	0.41–1.96	0.786
Baseline RBBB	0.93	0.13–6.83	0.942
Baseline PR ≥ 200 ms	1.70	0.63–4.62	0.296
Any pre-existing CD	2.02	0.90–4.50	0.086
Self-expanding valve	5.95	2.55–13.86	**<0.001**
ID_NCC_, per 1 mm	1.29	1.08–1.54	**0.005**
MSL, per 1 mm	0.90	0.71–1.14	0.382
MSL − ID_NCC_ < 0	2.14	1.00–4.61	0.051
Oversizing > 15%	6.92	2.40–19.93	**<0.001**
Tapered LVOT	9.73	1.19–79.67	**0.034**

ORs are expressed per 1-SD increase for biomarkers, per 1-year increase for age, per 1 mm increase for ID_NCC_ and MSL, and versus the reference category for categorical variables. SD values used for biomarker standardization were calculated from the analytic cohort and were as follows: CRP, 2.06 mg/L; ln(CAR), 0.8919; IL-6, 4.84 ng/L; PCT, 0.0193 µg/L; ferritin, 142.65 µg/L; LDH, 45.55 U/L; and TGF-β1, 3,156 pg/mL. Statistically significant *p*-values are shown in bold. Abbreviations: ln(CAR), natural logarithm of C-reactive protein-to-albumin ratio; CD, conduction disorder; CI, confidence interval; CRP, C-reactive protein; ID_NCC_, implantation depth at the non-coronary cusp; IL-6, interleukin-6; LDH, lactate dehydrogenase; LVOT, left ventricular outflow tract; MSL, membranous septum length; OR, odds ratio; PCT, procalcitonin; PR, PR interval; RBBB, right bundle branch block; SD, standard deviation; TGF-β1, transforming growth factor-β1.

**Table 7:** Biomarker-based clinical/procedural multivariable logistic regression models for new-onset conduction disorders after TAVI.

Model	Variable	OR	95% CI	*p*-Value
Model 1: IL-6	IL-6, per 1-SD	10.70	2.43–47.07	**0.002**
	Self-expanding valve	6.63	1.84–23.85	**0.004**
	ID_NCC_, per 1 mm	1.21	0.97–1.51	0.093
	Oversizing > 15%	2.34	0.59–9.24	0.226
	Any pre-existing CD	2.67	0.89–8.04	0.081
Model 2: CRP	CRP, per 1-SD	1.53	0.92–2.60	0.120
	Self-expanding valve	3.42	1.11–10.56	**0.033**
	ID_NCC_, per 1 mm	1.29	1.05–1.58	**0.016**
	Oversizing > 15%	4.25	1.16–15.51	**0.029**
	Any pre-existing CD	3.77	1.32–10.72	**0.013**
Model 3: ln(CAR)	ln(CAR), per 1-SD	1.52	0.93–2.51	0.098
	Self-expanding valve	3.39	1.10–10.44	**0.034**
	ID_NCC_, per 1 mm	1.31	1.06–1.62	**0.011**
	Oversizing > 15%	4.34	1.19–15.08	**0.026**
	Any pre-existing CD	3.64	1.27–10.40	**0.016**

ORs are expressed per 1-SD increase for biomarkers, per 1 mm increase for ID_NCC_, and versus the reference category for categorical variables. SD values used for biomarker standardization are provided in the Table 6. Statistically significant *p*-values are shown in bold. Abbreviations: ln(CAR), natural logarithm of C-reactive protein-to-albumin ratio; CD, conduction disorder; CI, confidence interval; CRP, C-reactive protein; ID_NCC_, implantation depth at the non-coronary cusp; IL-6, interleukin-6; OR, odds ratio; SD, standard deviation; TAVI, transcatheter aortic valve implantation.

**Table 8:** Receiver operating characteristic analysis of individual biomarkers for new-onset conduction disorders after TAVI.

Biomarker	AUC	95% CI	*p*-Value	Exploratory Cut-Off	Sensitivity (%)	Specificity (%)
CRP (mg/L)	0.617	0.514–0.721	**0.027**	1.7	58.6	63.0
CAR	0.617	0.513–0.720	**0.027**	0.038	56.9	64.8
IL-6 (ng/L)	0.730	0.637–0.823	**<0.001**	4.88	60.3	81.5
PCT (µg/L)	0.505	0.397–0.612	0.930	0.041	32.8	74.1
LDH (U/L)	0.581	0.475–0.686	0.134	169	77.6	38.9
Ferritin (µg/L)	0.474	0.367–0.582	0.641	10	100.0	3.7
TGF-β1 (pg/mL)	0.573	0.467–0.679	0.178	13,812.6	74.1	44.4

Exploratory cut-off values were determined using the Youden index and are presented descriptively for exploratory purposes only; they were not developed or validated as clinically applicable thresholds. Statistically significant *p*-values are shown in bold. Abbreviations: AUC, area under the receiver operating characteristic curve; CAR, C-reactive protein-to-albumin ratio; CI, confidence interval; CRP, C-reactive protein; IL-6, interleukin-6; LDH, lactate dehydrogenase; PCT, procalcitonin; TAVI, transcatheter aortic valve implantation; TGF-β1, transforming growth factor-β1.

**Table 9:** Exploratory univariable logistic regression analysis of echocardiographic parameters evaluated for association with new-onset conduction disorders after TAVI.

Echocardiographic Parameter	OR	95% CI	*p*-Value
LV EF, %	0.95	0.65–1.38	0.784
LV SVi, mL/m^2^	0.63	0.43–0.94	**0.022**
GLS, %	0.74	0.50–1.08	0.117
Basal GLS, %	0.57	0.38–0.86	**0.007**
AB strain ratio ≥ 2	5.73	2.49–13.17	**<0.001**
EF/GLS ratio	1.58	1.03–2.41	**0.034**
EF/basal GLS ratio	1.86	1.13–3.07	**0.015**
RASP > 1	4.76	1.48–15.34	**0.009**
AS-related cardiac damage stage	1.72	1.08–2.73	**0.023**

ORs for continuous variables are expressed per one standard deviation increase and versus the reference category for categorical variables. GLS values are absolute values. Statistically significant *p*-values are shown in bold. Abbreviations: AB, apical-to-basal; AS, aortic stenosis; CI, confidence interval; EF, ejection fraction; GLS, global longitudinal strain; LV, left ventricular; OR, odds ratio; RASP, relative apical sparing pattern; SVi, stroke volume index; TAVI, transcatheter aortic valve implantation.

**Table 10:** Exploratory biomarker–echocardiographic multivariable logistic regression models for new-onset conduction disorders after TAVI.

Model	Variable	OR	95% CI	*p*-Value
IL-6 + ECHO	IL-6, per 1-SD	9.08	2.47–33.30	**0.001**
	LV SVi	0.91	0.56–1.49	0.709
	AB strain ratio ≥ 2	4.12	1.38–12.33	**0.011**
	EF/basal GLS ratio	1.32	0.73–2.38	0.358
	RASP > 1	1.17	0.27–5.07	0.837
CRP + ECHO	CRP, per 1-SD	1.70	1.03–2.83	**0.039**
	LV SVi	0.79	0.50–1.27	0.332
	AB strain ratio ≥ 2	4.90	1.73–13.94	**0.003**
	EF/basal GLS ratio	1.12	0.65–1.93	0.690
	RASP > 1	1.70	0.42–6.81	0.454
ln(CAR) + ECHO	ln(CAR), per 1-SD	1.59	0.99–2.55	0.055
	LV SVi	0.80	0.50–1.28	0.351
	AB strain ratio ≥ 2	5.13	1.89–13.89	**0.001**
	EF/basal GLS ratio	1.12	0.65–1.93	0.695
	RASP > 1	1.68	0.42–6.75	0.463

ORs are expressed per 1-SD increase for continuous variables and versus the reference category for categorical variables. SD values used for biomarker standardization are provided in the Table 6. GLS values are absolute values. Statistically significant *p*-values are shown in bold. Abbreviations: AB, apical-to-basal; ln(CAR), natural logarithm of C-reactive protein-to-albumin ratio; CI, confidence interval; CRP, C-reactive protein; ECHO, echocardiographic model; EF, ejection fraction; GLS, global longitudinal strain; IL-6, interleukin-6; LV SVi, left ventricular stroke volume index; OR, odds ratio; RASP, relative apical sparing pattern; SD, standard deviation; TAVI, transcatheter aortic valve implantation.

## Data Availability

Dataset available on request from the authors.

## Supplementary Materials

The following supporting information can be downloaded at: https://www.mdpi.com/article/10.3390/medicina62091623/s1, Table S1: Group comparisons and sensitivity analysis of the association between IL-6 and permanent pacemaker implantation after TAVI; Table S2: Model fit, calibration, and discrimination of biomarker-based clinical/procedural multivariable logistic regression models for new-onset conduction disorders after TAVI; Table S3: Comparison of the reference clinical/procedural model with the reference model additionally including IL-6 for new-onset conduction disorders after TAVI; Table S4: Sensitivity analysis of the IL-6 association with new-onset conduction disorders using natural log-transformed IL-6; Table S5: Sensitivity analysis of the IL-6 association with new-onset conduction disorders additionally adjusted for natural log-transformed NT-proBNP; Table S6: DeLong pairwise comparisons of ROC curves for biomarker-based clinical/procedural multivariable logistic regression models for new-onset conduction disorders after TAVI; Table S7: Model fit, calibration, and discrimination of exploratory biomarker–echocardiographic multivariable logistic regression models for new-onset conduction disorders after TAVI; Table S8: DeLong pairwise comparisons of ROC curves for exploratory biomarker–echocardiographic multivariable logistic regression models for new-onset conduction disorders after TAVI.

## Abbreviations

The following abbreviations are used in this manuscript:

AB	apical-to-basal
AS	aortic stenosis
AV	aortic valve
BEV	balloon-expandable valve
CAR	C-reactive protein-to-albumin ratio
CAVB	complete atrioventricular block
CD	conduction disorder
CRP	C-reactive protein
CS	conduction system
ECG	electrocardiography/electrocardiogram
ECHO	echocardiographic model
EF	ejection fraction
ELISA	enzyme-linked immunosorbent assay
GLS	global longitudinal strain
HAVB	high-degree atrioventricular block
ID_LCC_	implantation depth at the left coronary cusp
ID_NCC_	implantation depth at the non-coronary cusp
IL-6	interleukin-6
LAFB	left anterior fascicular block
LBBB	left bundle branch block
LDH	lactate dehydrogenase
LV	left ventricular/left ventricle
LVOT	left ventricular outflow tract
MSL	membranous septum length
NYHA	New York Heart Association
PCT	procalcitonin
PPI	permanent pacemaker implantation
RASP	relative apical sparing pattern
RBBB	right bundle branch block
RV	right ventricular
SEV	self-expanding valve
TAVI	transcatheter aortic valve implantation
TGF-β1	transforming growth factor-β1
THV	transcatheter heart valve
TTE	transthoracic echocardiography
VARC-3	Valve Academic Research Consortium 3
