# Supplementary material for: Preprocedural Biomarkers of Inflammation and Fibrosis and Echocardiographic Markers of Myocardial Remodeling in New-Onset Conduction Disorders After Transcatheter Aortic Valve Implantation
Source: Medicina (Kaunas). 2026 Aug 23;62(9):1623. doi: 10.3390/medicina62091623 (PMC13608881; doi:10.3390/medicina62091623)
Supplement: Supplementary file 1 [file medicina-62-01623-s001.zip › medicina-4496895-supplementary.pdf]

Supplementary Materials

Clinical, Biomarker-Based, Echocardiographic, and Sensitivity Analyses of Conduction Outcomes after TAVI

This file contains Tables S1–S8.

Table S1. Group comparisons and sensitivity analyses of the association between IL-6 and permanent pacemaker implantation after TAVI.

Analysis	Estimate / comparison	No PPI (n = 94)	PPI (n = 18)	Effect estimate	95% CI	*p*-value
IL-6, ng/L	Group comparison	3.91 (3.01–5.93)	5.09 (3.18–6.29)	—	—	0.438
IL-6, per 1-SD increase	Univariable logistic regression	—	—	OR 1.06	0.67–1.67	0.812
IL-6	Discrimination for PPI	—	—	AUC 0.578	0.41–0.70	—

IL-6 values are presented as median (IQR) and compared between groups using the Mann–Whitney U test. Logistic regression results are expressed as ORs per 1-SD increase in IL-6. Discrimination was assessed using the AUC. Statistically significant *p*-values are shown in bold.

Abbreviations: AUC, area under the receiver operating characteristic curve; CI, confidence interval; IL-6, interleukin-6; IQR, interquartile range; OR, odds ratio; PPI, permanent pacemaker implantation; SD, standard deviation; TAVI, transcatheter aortic valve implantation.

Table S2. Model fit, calibration, and discrimination of biomarker-based clinical/procedural multivariable logistic regression models for new-onset conduction disorders after TAVI.

Model	χ^2	Model *p*	Nagelkerke R^2	HL *p*	Accuracy	AUC (95% CI)
IL-6 model	50.97	**<0.001**	0.503	0.227	75.9	0.863 (0.784–0.922)
CRP model	37.88	**<0.001**	0.395	0.344	75.0	0.819 (0.733–0.886)
ln(CAR) model	38.01	**<0.001**	0.396	0.629	72.2	0.820 (0.734–0.887)

Model fit was assessed using the model χ^2 test and Nagelkerke R^2; calibration was assessed using the Hosmer–Lemeshow test, and discrimination was assessed using the AUC. Accuracy is expressed as a percentage. Statistically significant model *p* values are shown in bold.

Abbreviations: AUC, area under the receiver operating characteristic curve; CAR, C-reactive protein-to-albumin ratio; CI, confidence interval; CRP, C-reactive protein; HL, Hosmer–Lemeshow; IL-6, interleukin-6; ln(CAR), natural logarithm of the C-reactive protein-to-albumin ratio; TAVI, transcatheter aortic valve implantation.

Table S3. Comparison of the reference clinical/procedural model with the reference model additionally including IL-6 for new-onset conduction disorders after TAVI.

Model	-2LL	Nagelkerke R^2	AUC (95% CI)	LRχ^2 vs reference	LR *p*	ΔAUC	DeLong *p*
Reference model	113.9	0.371	0.809 (0.722–0.878)	—	—	—	—
Reference model + IL-6	98.1	0.503	0.863 (0.784–0.922)	15.829	**<0.001**	0.054	**0.045**

The reference clinical/procedural model included self-expanding valve, ID_{NCC}, oversizing >15%, and any pre-existing conduction disorder. The likelihood-ratio test compares the reference model with the reference + IL-6 model. DeLong's test compares the AUCs of the two models.

Abbreviations: −2LL, −2 log-likelihood; AUC, area under the receiver operating characteristic curve; CI, confidence interval; ID_{NCC}, implantation depth at the non-coronary cusp; IL-6, interleukin-6; LR, likelihood-ratio; TAVI, transcatheter aortic valve implantation.

Table S4. Sensitivity analysis of the IL-6 association with new-onset conduction disorders using natural log-transformed IL-6.

Variable	Adjusted OR	95% CI	*p*-value
ln(IL-6), per 1-SD	3.20	1.60–6.41	**0.001**
Self-expanding valve	6.94	1.94–24.77	**0.003**
ID_{NCC}, per 1-mm	1.22	0.97–1.52	0.083
Oversizing >15%	2.44	0.63–9.53	0.199
Any pre-existing conduction disorder	2.73	0.91–8.15	0.073

IL-6 was natural-log transformed and standardized before model inclusion. ORs are expressed per 1-SD increase for ln(IL-6), per 1-mm increase for ID_{NCC}, and versus the reference category for categorical variables. Statistically significant *p*-values are shown in bold.

Abbreviations: CI, confidence interval; ID_{NCC}, implantation depth at the non-coronary cusp; IL-6, interleukin-6; ln(IL-6), natural logarithm of interleukin-6; OR, odds ratio; SD, standard deviation.

Table S5. Sensitivity analysis of the IL-6 association with new-onset conduction disorders additionally adjusted for natural log-transformed NT-proBNP.

Variable	Adjusted OR	95% CI	*p*-value
IL-6, per 1-SD	7.86	1.81–34.21	**0.006**
Self-expanding valve	6.65	1.85–23.87	**0.004**
ID_{NCC}, per 1-mm	1.25	0.99–1.58	0.060
Oversizing >15%	2.54	0.63–10.25	0.189
Any pre-existing conduction disorder	2.3	0.75–7.06	0.144
ln(NT-proBNP), per 1-SD	1.43	0.86–2.36	0.169

ORs are expressed per 1-SD increase for biomarkers, per 1-mm increase for ID_{NCC}, and versus the reference category for categorical variables. SD values used for biomarker standardization are provided in the Table 6 footnote. Statistically significant *p*-values are shown in bold. NT-proBNP was natural-log transformed.

Abbreviations: CI, confidence interval; ID_{NCC}, implantation depth at the non-coronary cusp; IL-6, interleukin-6; ln(NT-proBNP), natural logarithm of N-terminal pro-B-type natriuretic peptide; OR, odds ratio; SD, standard deviation.

Table S6. DeLong pairwise comparisons of ROC curves for biomarker-based clinical/procedural multivariable logistic regression models for new-onset conduction disorders after TAVI.

Comparison	ΔAUC	95% CI	*p*-value
IL-6 model vs. CRP model	0.044	−0.001 to 0.089	0.056
IL-6 model vs. ln(CAR) model	0.043	−0.001 to 0.087	0.054
CRP model vs. ln(CAR) model	0.001	−0.014 to 0.016	0.890

Pairwise comparisons of AUCs were performed using the DeLong test. ΔAUC represents the absolute difference between the AUCs of the compared models.

Abbreviations: AUC, area under the receiver operating characteristic curve; CAR, C-reactive protein-to-albumin ratio; CI, confidence interval; CRP, C-reactive protein; IL-6, interleukin-6; ln(CAR), natural logarithm of the C-reactive protein-to-albumin ratio; ROC, receiver operating characteristic; TAVI, transcatheter aortic valve implantation.

Table S7. Model fit, calibration, and discrimination of exploratory biomarker–echocardiographic multivariable logistic regression models for new-onset conduction disorders after TAVI.

Model	χ^2	Model *p*	Nagelkerke R^2	HL *p*	Accuracy	AUC (95% CI)
IL-6 + ECHO model	40.47	**<0.001**	0.405	0.220	75.0	0.811 (0.726–0.879)
CRP + ECHO model	28.78	**<0.001**	0.302	0.558	72.3	0.773 (0.684–0.847)
ln(CAR) + ECHO model	29.65	**<0.001**	0.310	0.516	68.8	0.783 (0.695–0.855)

Model fit was assessed using the model χ^2 test and Nagelkerke R^2; calibration was assessed using the Hosmer–Lemeshow test, and discrimination was assessed using the AUC. Accuracy is expressed as a percentage. Statistically significant model *p* values are shown in bold.

Abbreviations: AUC, area under the receiver operating characteristic curve; CAR, C-reactive protein-to-albumin ratio; CI, confidence interval; CRP, C-reactive protein; ECHO, echocardiography; HL, Hosmer–Lemeshow; IL-6, interleukin-6; ln(CAR), natural logarithm of the C-reactive protein-to-albumin ratio; TAVI, transcatheter aortic valve implantation.

Table S8. DeLong pairwise comparisons of ROC curves for exploratory biomarker–echocardiographic multivariable logistic regression models for new-onset conduction disorders after TAVI.

Comparison	ΔAUC	95% CI	*p*-value
IL-6 + ECHO model vs. CRP + ECHO model	0.038	−0.028 to 0.104	0.256
IL-6 + ECHO model vs. ln(CAR) + ECHO model	0.028	−0.040 to 0.097	0.421
CRP + ECHO model vs. ln(CAR) + ECHO model	0.010	−0.016 to 0.036	0.458

Pairwise comparisons of AUCs were performed using the DeLong test. ΔAUC represents the absolute difference between the AUCs of the compared models.

Abbreviations: AUC, area under the receiver operating characteristic curve; CAR, C-reactive protein-to-albumin ratio; CI, confidence interval; CRP, C-reactive protein; ECHO, echocardiography; IL-6, interleukin-6; ln(CAR), natural logarithm of the C-reactive protein-to-albumin ratio; ROC, receiver operating characteristic; TAVI, transcatheter aortic valve implantation.
